# The role of NINJ1 in diseases

**DOI:** 10.1038/s41420-026-03064-4

**Published:** 2026-03-26

**Authors:** Shijun Bao, Fengxu Chen, Ziyi Guo, Wen Ding, Fu Gao, Jiaming Guo

**Affiliations:** Department of Radiation Medicine, College of Naval Medicine, Naval Medical University, Shanghai, China

**Keywords:** Cell biology, Molecular biology

## Abstract

Nerve injury-induced protein 1 (NINJ1) is a multifunctional membrane protein historically studied for its roles in nerve regeneration and cell adhesion. A groundbreaking study fundamentally revised our understanding by demonstrating that NINJ1 acts as the active executor of plasma membrane rupture in lytic cell death pathways such as pyroptosis and ferroptosis, establishing this final step as a biologically regulated process. Recent structural insights now reveal that NINJ1 adopts distinct molecular forms—including the full-length monomer, a soluble fragment, and a membrane-rupturing oligomer—which dictate its functional roles in adhesion, chemotaxis, and cell lysis. This revised understanding calls for a systematic integration of previous observations, particularly given NINJ1’s context-dependent and often contradictory roles in inflammation, cancer, and tissue injury. Here, we review the structural basis of NINJ1 function, its pathological implications, and propose a unified structure-function model to reconcile its diverse phenotypes and bridge its traditional roles with its newly identified function in membrane rupture.

## Facts

- NINJ1 is a member of NINJ family, involved in several cellular functions.
- NINJ1 serves as the primary executor of plasma membrane rupture in nearly all forms of lytic cell death.
- The structural plasticity of NINJ1 underpins its functional diversity, dictating whether it mediates homophilic adhesion, acts as a soluble chemokine-like fragment, or executes plasma membrane rupture.

## Open questions

- Does the newly established role of NINJ1 in PMR reconcile its previously observed, and often contradictory, roles in nerve regeneration, angiogenesis, and cancer?
- Can NINJ1, as a common final step in lytic death, serve as a unifying node to simplify the increasingly complex classification of cell death modalities?
- What are the specific ligands or protein interactions that trigger the conformational switch of NINJ1 from an autoinhibited dimer to a membrane-rupturing oligomer?
- What upstream signals and contextual cues determine the functional fate of NINJ1, steering it toward adhesion, chemotaxis, or plasma membrane rupture?
- If NINJ1 acts as a signaling messenger not an executor, does it utilize a novel structural form or operate within the known modes of action (homophilic adhesion, sNINJ1, or oligomerization)?

## Introduction

The plasma membrane separates life from the external environment, serving not only as a physical barrier but also as a dynamic interface that maintains cellular integrity, facilitates selective exchange, and coordinates signal transduction [1]. Its structural and functional preservation is indispensable for cellular homeostasis, and consequently, its irreversible disruption has long been equated with cellular demise [2]. For decades, the terminal event of plasma membrane rupture (PMR) was predominantly viewed as a passive, physical consequence of catastrophic homeostasis collapse [3–5]. Although proteins like GSDMs and MLKL actively form membrane pores that increase permeability, lead to ion imbalance and cell swelling, and allow selective release of molecules like IL-1β, the complete rupture of the membrane and release of large proteins like lactate dehydrogenase (LDH) and HMGB1 were still attributed to a passive physical process, analogous to a balloon popping from overinflation [4, 6, 7].

However, in 2021, a groundbreaking research firstly report that NINJ1 is an active executioner of PMR, which fundamentally overturns the long-held perception of PMR as a passive process, revealing that the final event in lytic cell death, including pyroptosis, necroptosis, and ferroptosis, is still a precisely active biological process [8]. First identified in 1996, when scientists observed its upregulation in neurons and Schwann cells surrounding the distal nerve segment following axonal injury, NINJ1 was a membrane protein that belongs to the NINJ protein family together with NINJ2. This protein facilitates nerve regeneration and is also expressed in non-neuronal tissues, notably epithelial cells [9]. Although some studies have established strong correlations and causal links between NINJ1 and pathological injuries, the precise molecular mechanism by which it functions remains an unsolved mystery [9–12]. Key questions revolve around NINJ1’s mechanism of action: does it act as an enzyme (such as a kinase or ubiquitin ligase), a scaffolding protein, or does it exert its effects by regulating the activity or localization of other molecules?

NINJ1 exhibits striking functional pleiotropy, which underscores the critical importance of its structural plasticity. The protein can, on one hand, mediate pro-inflammatory lytic cell death via PMR, while on the other hand, it has been implicated in promoting neural tissue regeneration, suggesting a dual role in both destruction and repair [9]. While tissue-layer-specific and cell-type-specific regulations offer a plausible starting point for interpreting these discrepancies [13, 14], a more unified mechanism remains to be fully elucidated. This context-dependent nature extends to the immune system. In macrophages, NINJ1 exhibits a pro-inflammatory function by influencing M1/M2 polarization, yet its knockout in myeloid cells can yield an overall anti-inflammatory outcome by limiting the release of damage-associated molecular patterns (DAMPs) like HMGB1 and the formation of neutrophil extracellular traps [8, 15, 16]. The most striking illustration of this paradox is observed in infection models: NINJ1 deficiency increases susceptibility to a specific enteric pathogen, Citrobacter rodentium, while conversely proving protective in systemic sepsis models by attenuating overwhelming inflammation [17]. We propose that its diverse biological outcomes are dictated by distinct molecular forms: the full-length monomer at the membrane likely mediates homophilic adhesion via its N-terminal adhesion motif; upon cleavage by proteases like MMP9, a soluble fragment may act as a chemotactic signal in the extracellular space; and finally, its activated oligomeric form executes PMR. Thus, the apparent context-dependent duality of NINJ1—spanning adhesion, migration, and lysis—finds a unified explanation through its structural versatility.

This review aims to synthesize the evolving narrative of NINJ1, a cornerstone of the lytic cell death pathway, which fundamentally transformed our understanding of PMR from a passive endpoint to an actively regulated process. In an era of increasingly granular cell death nomenclature, any given pathological injury is rarely attributable to a single cell death mechanism alone, while the binary lytic/non-lytic framework stands out for its integrative power in explaining complex tissue damage, and NINJ1 sits at its very core. The distinctive contribution of this work lies in its synthesis of the often-overlooked pre-2021 research with the post-PMR revelation. This comprehensive perspective allows us to move beyond a singular focus on rupture and instead present a cohesive model: NINJ1’s celebrated context-dependent pleiotropy is governed by the function and transformation of its diverse molecular structures. Thus, our review provides the essential conceptual link that unites decades of phenotypic observations and recent groundbreaking research into a single, mechanistically grounded narrative, offering a definitive resource for the field.

## The structure and function of NINJ1

### Structural feature of NINJ1

**Fig. 1:**
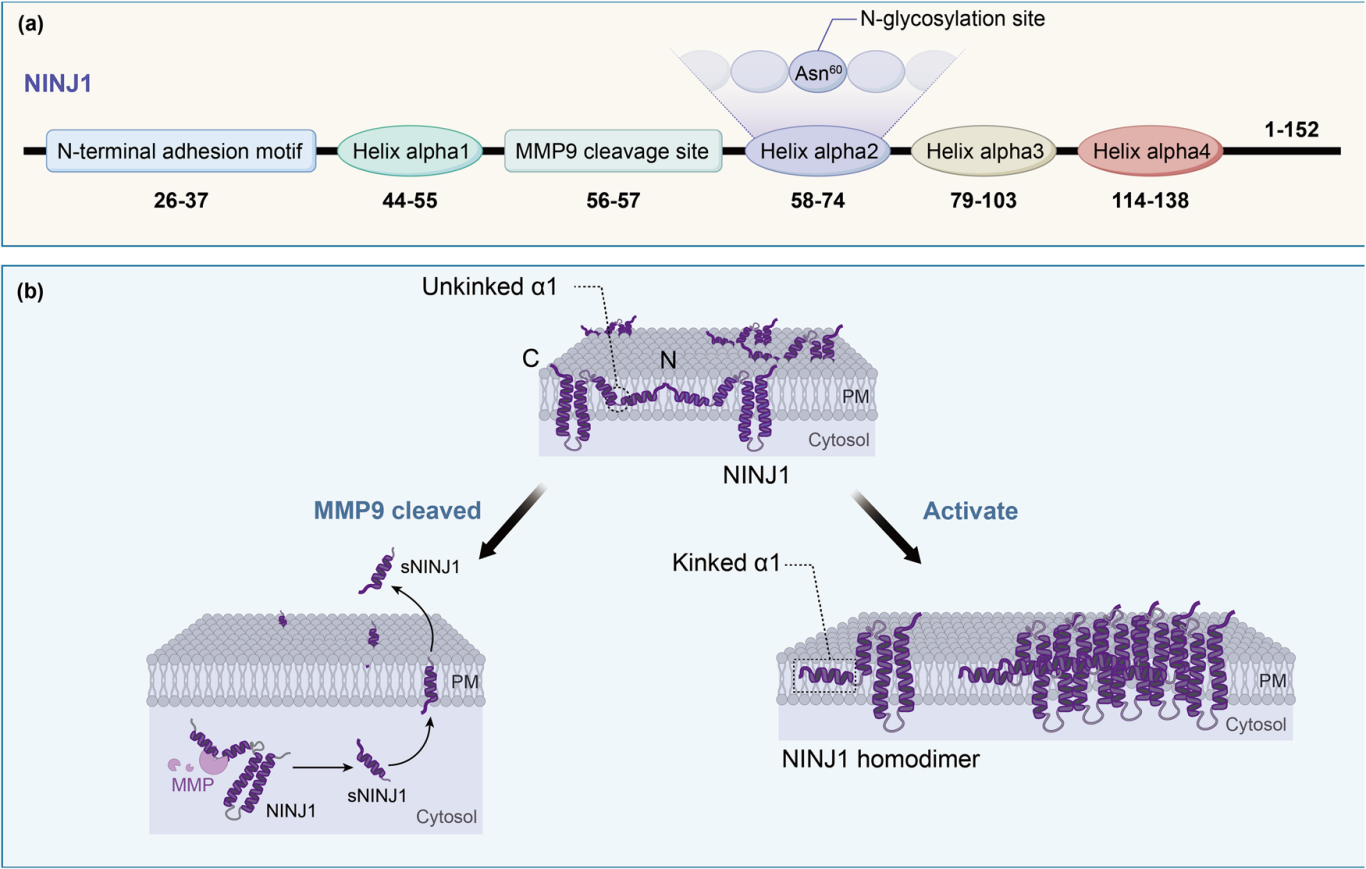
Molecular architecture and functional forms of NINJ1. **a** A schematic of the NINJ1 primary structure, highlighting key domains and sites: the N-terminal adhesion motif, MMP-9 cleavage site, N-glycosylation site, and the α1-α4 helices that constitute its amphipathic interface. **b** NINJ1 functional pleiotropy is mediated by distinct molecular forms. In the resting state, NINJ1 exists as an auto-inhibited homodimer with unkinked α1 helix. Upon activation, it undergoes conformational switching to oligomerize into a large pore complex responsible for plasma membrane rupture. Alternatively, proteolytic cleavage by MMP-9 liberates an N-terminal soluble fragment (sNINJ1) that exhibits chemotactic activity.

NINJ1, a member of the NINJ family, is encoded on chromosome 9 (9q22) [18]. The human NINJ1 protein comprises 152 amino acids. Beyond its full-length form, NINJ1 undergoes proteolytic processing by Matrix metalloproteinase-9 (MMP-9). This cleavage occurs at a specific site between leucine 56 and 57, generating a N-terminal extracellular fragment—sNINJ1. Structurally, this liberated sNINJ1 fragment resembles known chemokines, such as CXCL8 and CCL2 [19]. Each NINJ1 monomer is organized into four α-helices: two amphipathic helices (α1 and α2) and two transmembrane helices (α3 and α4). The inactive NINJ1 exists as a homodimer adopting a three-helix conformation with an unkinked α1. Upon activation, the dimer dissociates concomitant with α1 kinking, triggering a conformational transition to the four-helix active state—a structural rearrangement that hallmarks NINJ1 activation [20]. The active NINJ1 undergoes oligomerization to form complexes that directly mediate PMR [8]. This oligomerization process is critically dependent on N-glycosylation, as evidenced by studies where pharmacological inhibition with *tunicamycin* or point mutation of the glycosylation site (Asn⁶⁰→Gln/Ala) abrogates oligomerization [21] (Fig. 1a).

**Fig. 2:**
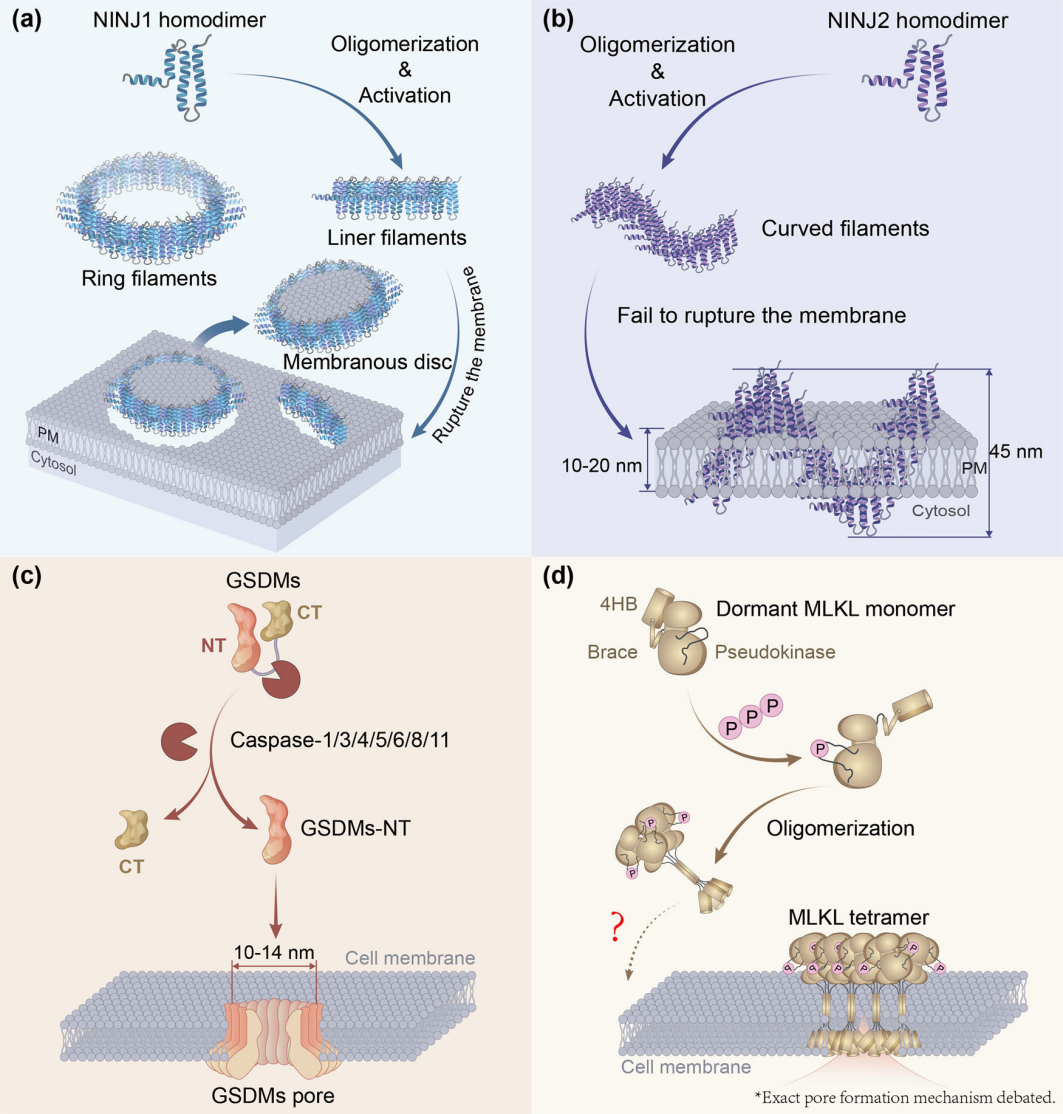
Comparative mechanisms of membrane disruption by NINJ1 and related pore-forming proteins. **a** Activated NINJ1 monomers oligomerize into large ring-shaped complexes that function as ring filaments to execute wholesale plasma membrane rupture. **b** Structural variations cause NINJ2 to form curved oligomers that lack membrane-disrupting capability. **c** Gasdermin N-terminal fragments assemble into ~10–14 nm pores that permit small molecule flux but restrict large DAMP release. **d** Phosphorylated MLKL tetramers mediate membrane permeabilization through heterogeneous disruptions rather than defined pores. The precise mechanism by which MLKL oligomers lead to membrane disruption remains an active area of investigation and is currently debated in the field (indicated by the dashed line and question mark in the panel). In contrast, the mechanisms for NINJ1, NINJ2, and GSDMs are more definitively established.

Intriguingly, NINJ2 is a paralog protein with 52% sequence identity to NINJ1, while it cannot mediate PMR [22]. This functional divergence stems from subtle structural differences between the two proteins: NINJ1 oligomers assemble into linear or ring filaments, whereas NINJ2 forms curved filaments. The inwardly curved architecture of NINJ2 oligomers prevents their organization into closed rings or stable assemblies at the plasma membrane, thus impairing membrane-disrupting capacity. Mutagenesis studies substituting NINJ2 residues with NINJ1 counterparts partially confer membrane-disrupting capacity to NINJ2 mutants, supporting this mechanism [23]. However, recent research indicates the core oligomerization machinery of NINJ2 is not intrinsically incompatible with lytic function, as transplanting the N-terminal domain of NINJ1 into NINJ2 is sufficient to confer membrane rupture activity to the latter [24]. This suggests that the key functional divergence may lie not in the capacity for oligomerization, but rather in the bending angle between adjacent oligomers, governed by their N-terminal, and consequently, the resulting dimensions of the entire oligomer relative to the membrane. Specifically, the NINJ2 oligomer exhibits an excessively large bending angle, resulting in the formation of a coil lying flat on the membrane with a diameter of approximately 45 nm. Given that the average thickness of the membrane is around 20 nm, this configuration causes NINJ2 to repeatedly traverse the membrane, yet it fails to excise a complete membrane segment [23] (Fig. 2b). The N-terminal of NINJ1 exerts its lytic function after oligomerization, whereas the N-terminal of NINJ2 may possess distinct functional properties. It is plausible to speculate that the N-terminal of NINJ2 may enable it to perform lytic functions in specific cellular contexts. For example, it has been proposed that the curved filamentous structure of NINJ2 might mediate membrane segment partitioning in cells with highly curved or tubular membranes, such as glial processes or neuronal axons. Furthermore, NINJ2 might interact with NINJ1 via its N-terminal, or incorporate into NINJ1 filaments to thereby inhibit NINJ1’s PMR function [23].

### Function of NINJ1

#### Role in PMR

**Fig. 3:**
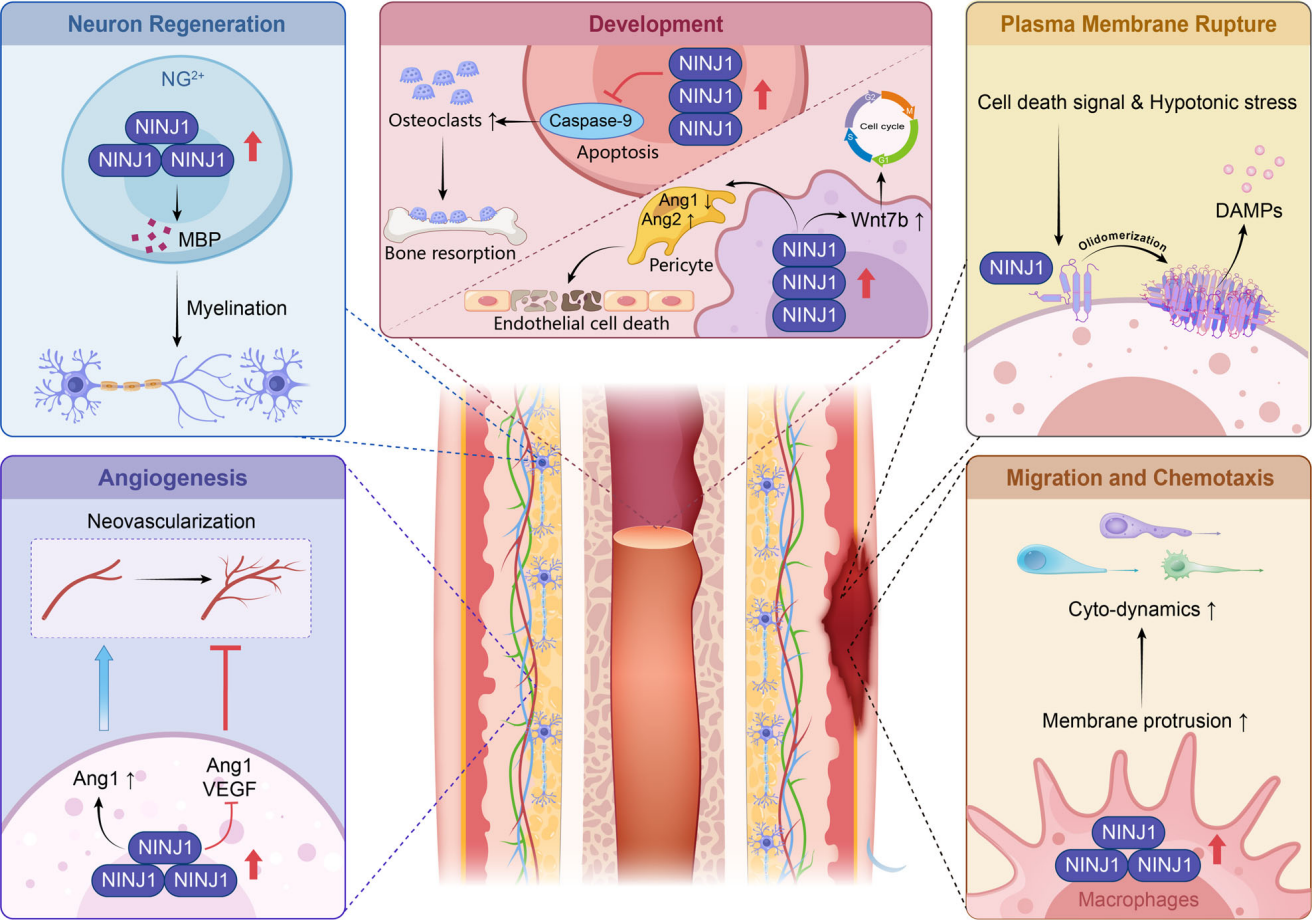
The physiological functions of NINJ1. This schematic summarizes the diverse biological roles of NINJ1, highlighting its context-dependent actions. NINJ1 mediates plasma membrane rupture as the terminal executor in lytic cell death, while in neural and vascular contexts, it supports regeneration and bidirectionally regulates angiogenesis. During development, it facilitates tissue remodeling and cell survival. Additionally, adhesion motif and sNINJ1 that directs leukocyte migration and chemotaxis. The functional versatility of NINJ1 across adhesion, inflammation, and repair processes base on the molecular architecture.

The plasma membrane demarcates the boundary between life and its environment, and its rupture signifies cellular demise. For a long time, scientists regarded the final rupture of the membrane as a passive event—an entropic process where cellular regulatory mechanisms become insufficient to maintain homeostasis and ultimately collapse. However, a seminal study from Vishva Dixit’s group (Nature, 2021) identified NINJ1 as the active executor of PMR during lytic cell death [8] (Fig. 3).

PMR represents the terminal event in lytic cell death, releasing DAMPs to propagate inflammation. Contrary to historical views of PMR as passive, NINJ1 deficiency (NINJ1^−/−^) in macrophages prevents PMR across pyroptotic, necroptotic, and apoptotic stimuli. NINJ1^−/−^ macrophages maintain a “ballooned morphology” with impaired release of LDH and DAMPs, establishing PMR as an NINJ1-dependent active process [8]. These findings indicate that PMR is an active process dependent on NINJ1.

NINJ1 regulates PMR through conformational switching between inactive dimers and active oligomers. NINJ1 adopts an autoinhibited, dimeric conformation, which is maintained by two distinct mechanisms: (1) the sequestration of the membrane-rupturing hydrophilic face within the dimeric interface prevents lipid contact; and (2) occlusion of the kinked-TM1 binding site on adjacent molecules suppresses spontaneous activation in the absence of stimuli [20]. Upon detection of cell death signals, the aforementioned conformational transition occurs, triggering NINJ1 oligomerization into the active form responsible for executing membrane rupture [3]. These activated NINJ1 oligomers then assemble into distinct circular structures on the plasma membrane, which are morphologically different from the pores formed by gasdermin proteins [25]. Subsequently, these oligomers disrupt membrane integrity through their amphipathic interface: the hydrophobic surfaces insert into the lipid bilayer, while the hydrophilic domains remain exposed extracellularly. Molecular dynamics simulations confirm that this configuration stabilizes the membrane edge via a “protective cap” mechanism, which nevertheless predisposes the membrane to rupture [26]. Furthermore, lipid staining experiments visually demonstrate that NINJ1 oligomers encircle membrane lesions, directly driving localized membrane loss and ultimate disintegration [25] (Fig. 1b).

The upstream signal for this auto-inhibited state was once thought to be various cell death signals, yet definitive molecular biological evidence has been lacking. Observers could only note the concurrent dissociation of the NINJ1 auto-inhibited dimer with the activation of cell death pathways. A hypothesis emerged suggesting that membrane swelling in the later stages of cell death might activate NINJ1, triggering its dissociation and conformational change [27]. Consistent with this model, macromolecular osmoprotectants (e.g., PEG4000) attenuate NINJ1-mediated PMR and mitigate inflammatory damage [14]. As research progressed, it was discovered that mechanical stretch alone—without activating other cell death programs—is sufficient to cause membrane rupture via NINJ1, establishing membrane tension itself as a direct upstream physical signal [28]. In pyroptosis, GSDMD-formed pores are insufficient for complete rupture; they require cellular swelling, which generates additional membrane tension, subsequently sensed by NINJ1 to execute the final rupture [28]. However, whether physical signals also activate the auto-inhibited dimer in other NINJ1-dependent cell death modalities remains to be fully investigated.

The engagement of NINJ1 as the terminal executor of PMR is not universal but exhibits remarkable context-dependence across different lytic death signals. This is most strikingly illustrated in ferroptosis, where the requirement for NINJ1 is inducer-specific. While its ablation protects against PMR induced by direct GPX4 inhibition (e.g., RSL3) [29], it is dispensable when ferroptosis is triggered via system Xc− inhibition (e.g., erastin) [30, 31]. This fundamental difference suggests that these inducers, despite converging on lipid peroxidation, engage distinct downstream effector pathways for terminal membrane disintegration [30]. Furthermore, certain forms of necroptosis can proceed to complete PMR independently of NINJ1, proving the existence of alternative execution pathways [32]. A point of convergence for several NINJ1-dependent pathways appears to be membrane perturbation and increased tension, as hypotonic stress alone is sufficient to trigger its oligomerization and PMR [14]. However, if NINJ1 were activated by a generic increase in membrane tension, it should be universally engaged during the terminal phase of all lytic deaths. To address this paradox, we propose that the mechanical cue sensed by NINJ1 may be specific. Diverse upstream death signals may be differentially integrated at the plasma membrane, with only some generating the specific mechanical cue that licenses NINJ1 oligomerization into its active form.

To fully appreciate the unique role of NINJ1, it is instructive to compare its mechanism with those of other well-characterized executors of lytic cell death (Fig. 2). The mechanism of the necroptotic executor MLKL remains debated. While it forms tetramers that translocate to and permeabilize the plasma membrane, evidence suggests it may cause more heterogeneous membrane disruptions rather than assembling into a uniform, defined pore like other pore-forming proteins [33] (Fig. 2d). This stands in clear contrast to the pyroptotic executor Gasdermin D (GSDMD), which unequivocally oligomerizes into structured pores of approximately 10–14 nm in diameter [34]. Although these GSDM pores permit the flux of ions and small cytokines like IL-1β, they function as a molecular sieve that cannot mediate the release of large DAMPs such as HMGB1 and LDH while NINJ1 can [8] (Fig. 2c). In pyroptosis, these GSDMD pores initiate a sequential cascade: by inducing osmotic imbalance and cell swelling, they generate the membrane tension required to activate NINJ1, which then executes the final, wholesale rupture [28]. By oligomerizing into a large permeabilization structure [23], responsible for wholesale PMR, NINJ1 creates an opening sufficiently large to facilitate the release of these bulk cellular contents, thereby defining itself as the last executor of terminal membrane disintegration [14, 32] (Fig. 2a). Notably, while NINJ1 is essential for PMR in pyroptosis and secondary necrosis, it is dispensable in apoptosis; its activation can be triggered by DAMPs including extracellular ATP, HMGB1, and IL-1β, underscoring its role as a regulated endpoint in inflammatory cell death [29].

#### Role in migration and chemotaxis

Directed cell migration is a fundamental biological process essential for development, immunity, and tissue repair [35]. While the core principles of migration and chemotaxis are well-established, the full repertoire of molecules orchestrating these steps, particularly at the interface of the plasma membrane and the cytoskeleton, remains incompletely defined [35]. Emerging evidence implicates NINJ1 as a potential player in this process [19, 36] (Fig. 3).

NINJ1 deficiency in bone marrow-derived macrophages reduces membrane protrusion formation and dynamics, impairing cellular motility. Concordantly, NINJ1 knockdown in RAW264.7 cells diminishes filopodia density, whereas its overexpression promotes protrusion formation and enhances cell migration. These findings establish NINJ1 as a regulator of membrane dynamics governing macrophage mobility and immune cell extravasation [37]. These observations provide strong phenotypic and gain/loss-of-function evidence linking NINJ1 to cell motility, although the precise molecular mechanism by which it regulates the cytoskeleton or membrane dynamics remains to be fully elucidated.

The role of NINJ1 in cell migration is functionally activated through proteolytic cleavage. MMP-9 processes NINJ1 to release sNINJ1. This fragment, which shares structural homology with chemokines like CXCL8 and CCL2, is directly responsible for mediating chemotactic activity, thereby providing a mechanistic link between NINJ1 and the regulation of cellular motility [19]. This represents a more direct mechanistic insight, identifying sNINJ1 and MMP-9. However, the specific receptor for sNINJ1 and the downstream signaling pathways it engages to direct chemotaxis are still undefined, representing a key area for future investigation.

#### Role in neurons regeneration

Functional recovery following nerve injury hinges on a complex sequence of events, including axonal regeneration, remyelination, and the reestablishment of synaptic connections [38]. While numerous molecules guide this process, the transmembrane protein NINJ1 has emerged as a significant player. Its established roles in homophilic adhesion position it as a potential key regulator in orchestrating structural and functional repair within the nervous system (Fig. 3).

NINJ1 was initially identified in neurons of the distal segment following nerve injury, where it promotes axonal regeneration [18]. Within the peripheral nervous system, Schwann cell precursors and perivascular cells (PCs)—both NG2-positive—critically depend on NINJ1. Genetic ablation of NINJ1 in NG2^+^ cells significantly reduces expression of myelin basic protein and diminishes the population of myelinated axons. This defect likely stems from the role of NINJ1 in mediating homophilic adhesion between neural cells [18], a process further supported by its co-localization and functional synergy with tight junction proteins. Notably, tight junction components are markedly downregulated in NINJ1-knockout systems [39]. Collectively, NINJ1 orchestrates peripheral nerve regeneration, primarily by governing myelination in NG2^+^ glial populations—including Schwann cell precursors and multipotent PCs [13].

The apparent contradiction between NINJ1-mediated neurite outgrowth [18] and its role in PMR may be resolved by distinct activation mechanisms. We propose that the homophilic adhesion underpinning nerve regeneration is primarily driven by the MMP-9-cleaved sNINJ1, functioning independently of NINJ1 oligomerization required for PMR. This model uncouples NINJ1’s reparative function from its lytic role, suggesting that context-dependent proteolysis and activation states enable its functional diversity in neuronal repair versus cell death.

#### Role in development

Embryonic development requires precisely coordinated processes ranging from programmed tissue remodeling to cell fate determination. Emerging evidence indicates that NINJ1 contributes to specific aspects of this complex program, particularly in contexts involving macrophage-mediated regression and osteoclast maturation (Fig. 3). However, the current evidence primarily describes phenotypic associations—such as its upregulation in macrophages during tissue regression, its pro-survival role in pre-osteoclasts, and its correlation with trophoblast dysfunction [40–43]—without establishing a direct link between its molecular structure and these diverse developmental functions.

To reconcile these observations mechanistically, we propose a hypothesis grounded in NINJ1’s structural versatility. The full-length membrane-bound form, via homophilic adhesion, could mediate critical cell-cell interactions, such as those stabilizing osteoclast precursors to promote survival [43] or facilitating macrophage adhesion during tissue remodeling [13]. Alternatively, context-dependent proteolysis by enzymes like MMP-9 may liberate the soluble sNINJ1 fragment, which could act as a paracrine or autocrine chemokine-like signal to recruit or modulate other cell types in developing tissues [19]. Finally, while its large oligomeric, pore-forming state is typically associated with pathological PMR, a tightly regulated, localized activation of this form cannot be entirely ruled out in extreme scenarios of developmental programmed cell clearance. This model shifts the focus from vague context-dependency to testable predictions: which molecular form of NINJ1 is present and necessary in each developmental context, and what are the specific proteolytic or activation cues that govern its form and function? Validating this hypothesis will require future studies combining cell type-specific genetic models with form-specific biochemical and imaging tools.

#### Role in angiogenesis

Angiogenesis is a highly coordinated process essential for development and tissue repair, requiring precise communication between endothelial cells and pericytes. Emerging evidence implicates NINJ1 as a context-dependent modulator within this complex interplay, particularly through its expression and function in pericytes. Rather than serving as a universal promoter or inhibitor, NINJ1 appears to fine-tune vascular remodeling by dynamically regulating key angiogenic factors and pericyte-endothelial interactions, ensuring balanced neovascularization (Fig. 3).

NINJ1 bidirectionally regulates vascular remodeling through dynamic modulation of angiogenic factors in pericytes. Downregulation of NINJ1 in pericytes significantly elevates production of vascular endothelial growth factor and angiopoietin-1 (Ang1), whereas its overexpression suppresses these factors, thereby attenuating pericyte-mediated trophic support and inhibiting neovascularization [44]. Paradoxically, NINJ1 can also function as a pro-angiogenic factor: its overexpression facilitates pericyte-endothelial cell interactions and upregulates angiopoietin-1 (Ang1) expression in pericytes [28]. We hypothesize that this cell-type-specific signaling output is influenced by differential receptor engagement, proteolytic processing (generating sNINJ1), or integration with other pathways. Thus, the “context” likely resides in the specific molecular interactors and modifiers present in a given vascular niche. Resolving this specificity is crucial for any attempt to therapeutically modulate NINJ1 in vascular diseases.

A critical pending question remains: what mechanisms determine the context-dependent pro- or anti-angiogenic role of NINJ1? Tissue-specific factors represent one compelling possibility. Exacerbated intimal hyperplasia, elevated vascular permeability, and enhanced macrophage infiltration with upregulated inflammatory cytokines in the adventitia were exhibited in NG2⁺ cell (pericytes/vascular smooth muscle cells)-specific NINJ1 knockout mice [13]. Divergent phenotypes across vascular compartments suggest potential tissue layer-specific regulatory mechanisms governing NINJ1 function. However, these in vivo genetic studies provide strong evidence for NINJ1’s functional importance in vascular homeostasis, yet they do not elucidate the underlying structural mechanisms.

## NINJ1 and diseases

### Inflammatory diseases

**Fig. 4:**
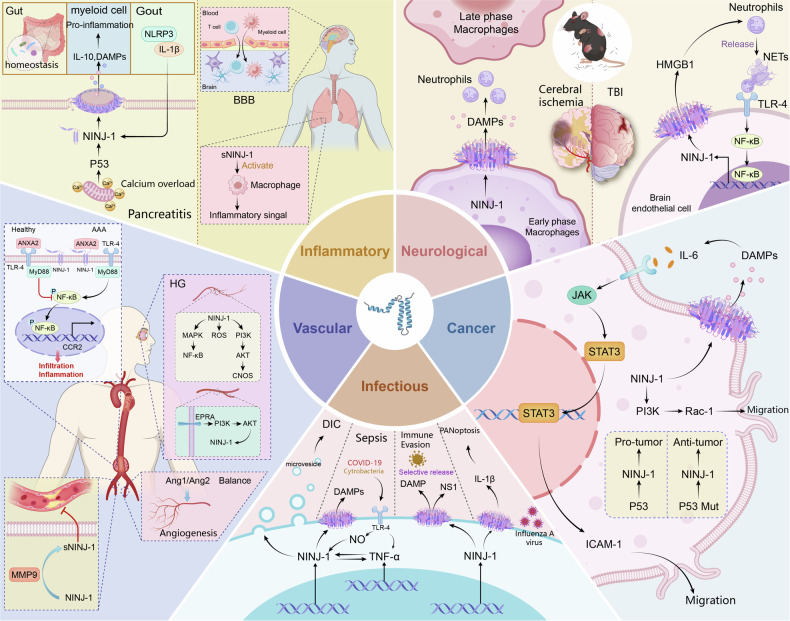
Context-dependent roles of NINJ1 across human pathologies. NINJ1 is implicated in a spectrum of human diseases, including inflammatory conditions, neurological and vascular injuries, infectious diseases, and cancer. A hallmark of its involvement is its context-dependent functional duality, where it can exert either detrimental or beneficial effects. This apparent paradox is resolved by its structural basis: the specific molecular form of NINJ1 engaged—governing adhesion, chemotaxis, or plasma membrane rupture—determines its ultimate role in disease pathogenesis.

Chronic inflammation underpins a diverse spectrum of human diseases, ranging from sterile injury to dysregulated autoimmunity (A detailed discussion of infection-triggered inflammatory responses is provided in a later, independent section) (Fig. 4).

In severe acute pancreatitis and gout, NINJ1 acts as the terminal executor of PMR, directly translating cellular injury into pro-inflammatory signaling. In severe acute pancreatitis, a specific calcium-p53-NINJ1 axis is activated, where calcium overload culminates in NINJ1-mediated acinar cell rupture [45]. Similarly, in gout, the formation of NLRP3 inflammasome and IL-1β release creates a feed-forward loop that upregulates NINJ1, ultimately leading to the lytic death of affected cells and the amplification of local inflammation [46]. In these models, NINJ1’s role is predominantly pro-inflammatory, serving as the critical link between initial damage and full-blown inflammatory pathology. The therapeutic efficacy of NINJ1 inhibition in these conditions underscores its non-redundant role as a gatekeeper of PMR. While the role of NINJ1 becomes intriguingly complex in inflammatory bowel disease, where it exhibits a stark paradoxical duality. Global NINJ1 deficiency exacerbates colitis, suggesting a protective, anti-inflammatory role, potentially through maintaining gut microbiota homeostasis [47]. Conversely, myeloid-specific NINJ1 deletion attenuates disease, revealing a pro-inflammatory function within the leukocyte [15, 48]. This paradox highlights that NINJ1’s net effect depends on its cellular context: in non-hematopoietic cells (e.g., gut epithelium), it may uphold barrier integrity, while in infiltrating myeloid cells, it drives pathology through PMR and potentially by influencing macrophage polarization. Given that macrophage polarization is highly influenced by cytokine signals from the microenvironment [49], this raises a compelling hypothesis: could the PMR of a subset of macrophages itself release signals (e.g., IL-10) that skew the remaining macrophages toward an M2, reparative phenotype? This would position NINJ1 not just as a killer, but as a subtle orchestrator of the immune landscape. This stark dichotomy underscores that the net pathological outcome is a sum of its compartmentalized actions. It further implies that therapeutic targeting of NINJ1 must be cell-type precise; systemic inhibition might harm epithelial repair while myeloid-specific blockade could be beneficial. This complexity positions NINJ1 not merely as a universal killer but as a nuanced modulator of tissue homeostasis.

In multiple sclerosis (MS), NINJ1 plays a distinct role by governing the trafficking of immune cells across the blood-brain barrier (BBB) [50]. Here, its classical function as a homophilic adhesion molecule is co-opted to facilitate the adhesion and trans-endothelial migration of both myeloid cells and T cells [11, 51]. This illustrates a PMR-independent, structural role for NINJ1 in inflammation, where it directly controls the recruitment of pathogenic lymphocytes into the central nervous system. Therapeutic blockade of NINJ1 reduces spinal cord leukocyte infiltration and ameliorates experimental autoimmune encephalomyelitis pathology [52]. Similarly, A paradigm shift is emerging with the discovery of the sNINJ1, which also uncouples NINJ1’s function from PMR. In idiopathic pulmonary fibrosis, recombinant sNINJ1 directly activates macrophages and drives pro-fibrotic inflammation [53]. This establishes a novel mechanism: NINJ1 can be cleaved to release a chemokine-like domain that acts as a diffusible inflammatory signal, amplifying immune responses without necessitating cell lysis. This “signaling” function vastly expands the potential scope of NINJ1’s involvement in chronic inflammatory and fibrotic diseases.

### Cardiovascular diseases

The integrity of the vasculature is compromised across a spectrum of diseases. NINJ1 emerges as a pivotal, yet paradoxical, regulator whose role shifts from pathogenic to protective depending on the cellular context, its molecular form, and the specific disease microenvironment (Fig. 4).

A clear dichotomy is observed between its roles in diabetic vascular complications versus atherosclerosis. In diabetic vasculopathy, NINJ1 acts as a pathogenic driver, exacerbating endothelial dysfunction through mechanisms involving oxidative stress and inflammatory pathway activation (e.g., p38 MAPK/NF-κB) while inhibiting protective signaling (e.g., PI3K/Akt/eNOS) [54, 55]. This detrimental role extends to diabetic retinopathy and erectile dysfunction, where targeting NINJ1 shows therapeutic potential [56–58].

Conversely, in atherosclerosis, a protective function is attributed specifically to the soluble fragment sNINJ1, generated by MMP-9 cleavage. This fragment inhibits monocyte recruitment and macrophage inflammation, demonstrating anti-atherosclerotic activity [36]. This stark contrast underscores that NINJ1’s functional output is not intrinsic but is defined by proteolytic processing and the resulting molecular form.

The cellular source is another critical determinant of function. In inflammatory contexts such as abdominal aortic aneurysm, macrophage-expressed NINJ1 promotes infiltration and disease progression via pathways like TLR4/NF-κB/CCR2 [59]. In contrast, pericyte-expressed NINJ1 is essential for vascular stability and repair, as its loss in hindlimb ischemia models impairs angiogenesis and exacerbates inflammation [60, 61]. Epidemiological studies have further associated NINJ1 expression with the risk of various arterial diseases (e.g., large-artery stroke, aortic dissection) [62–64], highlighting its clinical relevance while emphasizing the need to decipher the underlying cell-specific mechanisms.

In summary, NINJ1 in cardiovascular pathophysiology is not a unitary actor but a context-dependent node. Its impact—whether deleterious or beneficial—is dictated by a combination of factors: (1) the disease context (metabolic vs. atherosclerotic), (2) the proteolytic state (full-length protein vs. sNINJ1), and (3) the expressing cell type (endothelial cell, macrophage, or pericyte). Resolving how these variables integrate to steer NINJ1 function is crucial for developing targeted therapeutic strategies.

### Infectious diseases

Inflammation is a hallmark of infectious diseases. Upon sensing pathogen invasion, host cells activate NINJ1, which oligomerizes to actively execute the final disintegration of the plasma membrane. This ensures robust release of DAMPs, effectively initiating and amplifying the antibacterial inflammatory response while promoting antigen exposure. However, excessive activation of inflammation—as seen in sepsis—may lead to a systemic inflammatory response syndrome and exacerbate organ damage. Thus, the function of NINJ1 in infectious contexts is protective when appropriately regulated but becomes pathological when dysregulated (Fig. 4).

NINJ1^−/−^ mice exhibit high susceptibility to Citrobacter rodentium infection, revealing that PMR as a critical antimicrobial defense mechanism. By facilitating DAMPs release, NINJ1-mediated PMR restricts pathogen dissemination—demonstrating that cell lysis serves as an essential arm of innate immunity [65]. Furthermore, the Asp110Ala variant of NINJ1 confers a 2.42-fold increased risk of neurological damage in leprosy [66]. Recent research has revealed that NINJ1 mediates the final PMR and release of DAMPs during influenza A virus-induced PANoptosis, while partially regulating IL-1β secretion. Genetic ablation of NINJ1 significantly ameliorates pulmonary immunopathology and improves survival rates in mouse models [67].

Paradoxically, in sepsis models, NINJ1 inhibition attenuates systemic inflammation, reduces organ damage, and improves survival [17]. This protection reflects NINJ1’s dual pathology: it amplifies inflammation through TLR4-dependent mediators (TNF-α, NO) while exacerbating disseminated intravascular coagulation via leukocyte migration and platelet activation [17, 68, 69]. Molecularly, LPS directly binds NINJ1_(81-100aa)_ to trigger inflammatory signaling [69], whereas amlodipine indirectly suppresses NINJ1 expression via NF-κB inhibition, reducing monocyte adhesion [70]. Cell-type specificity is evident in acute liver failure, where global NINJ1 knockout—but not myeloid-specific ablation—alleviates TNF-α-induced hepatocyte apoptosis [71]. Once again, the association between NINJ1 and inflammation exhibits significant tissue specificity. Moreover, in severe infections like sepsis and COVID-19, systemic coagulopathy accompanies inflammation. Notably, NINJ1 is an essential driver of procoagulant microvesicle release during pyroptosis. Suppression of NINJ1 limited microvesicle and cytokine release, protecting mice from flagellin-induced DIC [72] (Fig. 4).

Beyond its endogenous role in regulating inflammation, NINJ1 can be co-opted by pathogens to facilitate infection, underscoring its function as a host defense factor is a double-edged sword. Specifically, murine norovirus hijacks NINJ1 oligomerization to selectively release both viral NS1 proteins and host DAMPs from speckle-like structures, thereby achieving immune evasion [73]. Concurrently, *Salmonella* exploits the NINJ1-related pathway to enhance its own intracellular replication [74].

Collectively, NINJ1 emerges as a multifaceted regulator of infection pathophysiology through PMR-dependent and independent mechanisms. The critical determinants of its net effect appear to be the anatomical localization and magnitude of its activation (localized vs. systemic) and the specific pathogen and host genetic background.

### Cancer

Three common hallmarks of cancer [75], including proliferation, metastasis, and tumor-promoting inflammation, are cellular processes in which the involvement of NINJ1 has been demonstrated (Fig. 4).

The most defining feature of NINJ1 in cancer is its functional duality, pivoted by p53 status. In models with wild-type p53, NINJ1 acts as a pro-tumorigenic factor, whereas in p53-deficient contexts, it can shift to exert tumor-suppressive effects [76]. This axis underscores that NINJ1 is not an intrinsic oncogene or tumor suppressor, but a context-dependent effector whose output is programmed by the genetic and signaling milieu. Moreover, radiation upregulates NINJ1 expression in a p53-dependent manner, establishing a feedback loop wherein NINJ1 itself contributes to p53 homeostasis [77, 78]. Within the tumor microenvironment, this activated p53-NINJ1 axis promotes endothelial-monocyte adhesion and monocyte infiltration, thereby driving pro-metastatic niche remodeling, a key mechanism underlying radiotherapy-associated recurrence [79].

Beyond p53, NINJ1’s role in cancer exhibits striking tissue and context specificity, leading to seemingly contradictory reports on its function in metastasis and its correlation with patient prognosis across different cancer types (Table 1). These observations can be critically integrated within the framework of NINJ1’s distinct molecular forms. The full-length adhesion-competent form may drive tumor cell migration and endothelial adhesion in certain contexts (e.g., in circulating tumor cells). The soluble sNINJ1 fragment could act as a paracrine signal within the tumor microenvironment, potentially influencing immune cell recruitment or angiogenesis. Finally, the oligomeric, pore-forming form may execute immunogenic cell death (e.g., pyroptosis) in response to therapy, shaping an inflammatory microenvironment. This “form-follows-function” model provides a mechanistic lens to reinterpret disparate phenotypic observations and generates testable hypotheses about which form is operational in specific tumor settings.

**Table 1:** The context-dependent roles of NINJ1 across various cancers.

Cancer type	Primary reported role of NINJ1	Mechanism	Prognostic correlation	References
Lung cancer	Pro-tumorigenic	•Negative regulator of migration via suppressing IL-6/STAT3 pathway.	Not specified	[103]
Prostate cancer	Pro-metastatic	•Upregulated in CTCs; promotes adhesion and motility	Not specified	[104]
Ovarian cancer	Tumor-suppressive	•Low expression correlates with advanced disease	Low expression → Poor survival	[105]
Retroperitoneal liposarcoma	Tumor-suppressive	•High expression associated with better outcome	High expression → Longer survival	[106]
Hepatocellular carcinoma	Dual	•Tissue upregulation •in vitro growth suppression via cell cycle arrest •Serum sNINJ1 as a biomarker	Serum sNINJ1 as diagnostic/prognostic marker	[10, 107, 108]
Colorectal cancer	Pro-tumorigenic	•Contributes to development in a testosterone-dependent manner	Not specified	[109]
Uveal melanoma	Pro-inflammatory	•Collaborates with GSDME to execute therapy-induced pyroptosis	Not specified	[110]
Leukemia	Pro-metastatic	•Mediates trans-endothelial migration via PI3K/Rac1 pathway	Not specified	[81]

### Neurological diseases

Neurological disorders encompass a wide spectrum of conditions, from neuropsychiatric abnormalities to acute brain injuries, often involving complex interactions between neuronal dysfunction and neuroinflammation. Within this landscape, NINJ1 emerges as a molecule of intriguing duality, participating in both the maintenance of central nervous system homeostasis and the execution of pathological processes. Evidence reveals that its absence under physiological conditions leads to significant behavioral deficits, while its targeted expression and activation during disease states critically contribute to neuroinflammatory cascades and cellular damage (Fig. 4).

In the intact central nervous system, NINJ1 serves a crucial, non-redundant role in maintaining synaptic homeostasis within corticothalamic circuits. Genetic ablation of NINJ1, either globally or specifically in neurons, results in compulsive grooming, self-injury, and anxiety-like behaviors [80]. Electrophysiological studies pinpoint the thalamus as a key site of dysfunction, revealing a paradox of glutamatergic dysregulation: despite decreased glutamate levels, there is an increase in ionotropic glutamate receptors alongside a reduction in functional synapses 69. This suggests that NINJ1 is essential for proper synaptic connectivity and glutamate receptor trafficking, with its loss leading to network instability that manifests as profound neuropsychiatric abnormalities.

In cerebral ischemia, NINJ1 exhibits a spatiotemporal functional duality, acting as a damaging agent early on and potentially facilitating repair later. Its expression dynamically shifts from an early phase in infiltrating neutrophils and endothelial cells to a later phase in reactive macrophages [12]. This temporal switch implies distinct roles: early NINJ1 may contribute to acute inflammation and cell death, while its later presence in macrophages could be linked to clearance of debris and tissue remodeling. Accordingly, therapeutic inhibition of NINJ1 via intranasal siRNA or the N-terminal competitor N-NAM during the acute phase robustly protects the brain by reducing neutrophil infiltration and shrinking infarct volume [81]. Mechanistically, N-NAM’s protective effect is linked to the activation of the pro-angiogenic Ang1-Tie2/AKT pathway [82], revealing a surprising role for NINJ1 signaling in coupling neuroinflammation to vascular repair processes.

In traumatic brain injury, NINJ1 assumes a distinctly pathological role as the terminal executor of a destructive neuroinflammatory feedback loop. Here, neutrophil-derived NETs initiate the cascade by inducing TLR4/NF-κB-mediated pyroptosis in brain endothelial cells. Within this pathway, NINJ1 activation is responsible for the final PMR, leading to HMGB1 release which, in turn, further amplifies NET formation [83]. This NINJ1-dependent cycle directly exacerbates BBB disruption, and its genetic ablation effectively breaks this loop, attenuating cerebrovascular leakage and improving outcomes [83]. This mechanism highlights NINJ1’s function as a critical amplifier of secondary injury in neurovascular damage.

## NINJ1-targeted therapies

Current therapeutic strategies targeting NINJ1 primarily focus on several key mechanisms: inhibiting its expression (as seen with natural compounds and miRNA), blocking its oligomerization (e.g., Glycine, Muscimol and neutralizing antibodies), and interfering with its mediated cell adhesion and migration (such as competitive peptide segments). It is particularly noteworthy that different strategies may actually target distinct functional forms of NINJ1: some primarily affect the generation and function of the soluble fragment sNINJ1, while others focus on regulating the oligomerization process of the membrane-bound form (Table 2).

**Table 2:** Targeting NINJ1: summary of current agents.

Drug type	Category	Mechanism	References
Natural compounds			
Arbutin	Natural polyphenols	•Inhibit the adhesion activity of BV2 cells •reduce the expression of NINJ1 •block the nuclear translocation and transcriptional activity of NF-κB	[84]
Chlorogenic acid	Natural polyphenols	•Reduce the adhesion ability of macrophages induced by LPS •down-regulate the expression of NINJ1 •Antioxidant and anti-obesity effects	[85]
Phaseolin	Flavonoid	•Reduce the activity of NINJ1 and MMP •inhibit the nuclear translocation of NF-κB •decrease the recruitment of white blood cells	[86]
Phenyl-β-D-Glucopyranoside	Glycoside	•Inhibit the expression of NINJ1 and MMP activity induced by endotoxin •anti-inflammatory and anti-cancer properties	[87]
AENJ	Marine natural products	•Reduce the expression of NINJ1 and the activity of MMP-2/MMP-9 •block the nuclear translocation of NF-κB	[88]
Glycine	Amino acid	•Inhibit the NINJ1 oligomerization	[91]
Vitamin D	Steroid	•NINJ1 is one of the primary targets of vitamin D and is regulated by the vitamin D receptor (VDR)	[89, 90]
Small molecule compounds			
DMHCA	Synthetic small molecules	•Forming a high-affinity binding with NINJ1	[92]
Cristacarpin	Isoprenylated dihydrochalcones	•Down-regulate the expression of NINJ1 and MMP •reduce macrophage adhesion	[93]
Muscimol	GABA receptor agonist	•Blockage of NINJ1 oligomerization •inhibit the final stage of pyroptosis where the plasma membrane ruptures	[94]
Antibodies and targeted peptides			
N-NAM Simulated Inhibitor	Competitive peptide segment	•Simulate the N-terminal adhesion motif of NINJ1 •inhibit the cell migration and inflammatory signaling mediated by NINJ1	[81]
ML56/PN12 Simulated Peptide	Competitive peptide segment	•Simulate sNINJ1 to inhibit the expression of pro-inflammatory genes in macrophages and the transendothelial migration of monocytes •alleviate atherosclerosis	[36]
Anti-NINJ1 monoclonal antibody	Neutralizing antibody	•Block the oligomerization of NINJ1 •promote nerve regeneration and angiogenesis	[57, 95, 96]
miR-125a-5p	microRNA	•Reduce the expression of NINJ1 to inhibit inflammation and the progression of diabetic retinopathy	[97]

However, despite the potent efficacy demonstrated by NINJ1 inhibitors in animal studies, preclinical and clinical trials for these drugs remain urgently needed. While targeting NINJ1 holds immense therapeutic promise across inflammatory, vascular, and oncological diseases, a critical appraisal of potential risks is imperative, given its multifaceted physiological roles. The most salient risk is the compromise of innate host defense. As highlighted earlier, NINJ1-mediated PMR is essential for containing pathogens like *Citrobacter rodentium* [66], and its systemic inhibition could increase susceptibility to infections. Conversely, in conditions like sepsis, where NINJ1 drives excessive inflammation, inhibition is beneficial [17]. This paradox underscores that the disease context and the timing of intervention are paramount in assessing risk-benefit ratios.

Furthermore, the functional pleiotropy of NINJ1, dictated by its distinct molecular forms, demands form-specific therapeutic strategies. A blanket inhibition of all NINJ1 functions is likely untenable. Instead, a precision medicine approach should be guided by the pathogenic form of NINJ1 predominant in a given disease: (1) Inhibiting the oligomeric pore-forming form (e.g., with Glycine, Muscimol, or neutralizing antibodies) is rational in pathologies driven by aberrant PMR, such as fulminant hepatitis, severe pancreatitis, or inflammatory tissue damage in sterile injury. (2) Modulating the soluble sNINJ1 fragment (e.g., by inhibiting its generation via MMP-9 or using mimetic peptides like ML56/PN12) may be preferable in chronic inflammatory or angiogenic diseases where its chemokine-like signaling perpetuates pathology, such as in atherosclerosis or fibrosis, while potentially sparing host defense. (3) Blocking the adhesive function of full-length NINJ1 (e.g., with competitive peptides like N-NAM) could target leukocyte trafficking in diseases like multiple sclerosis without directly inducing cell lysis.

**Fig. 5:**
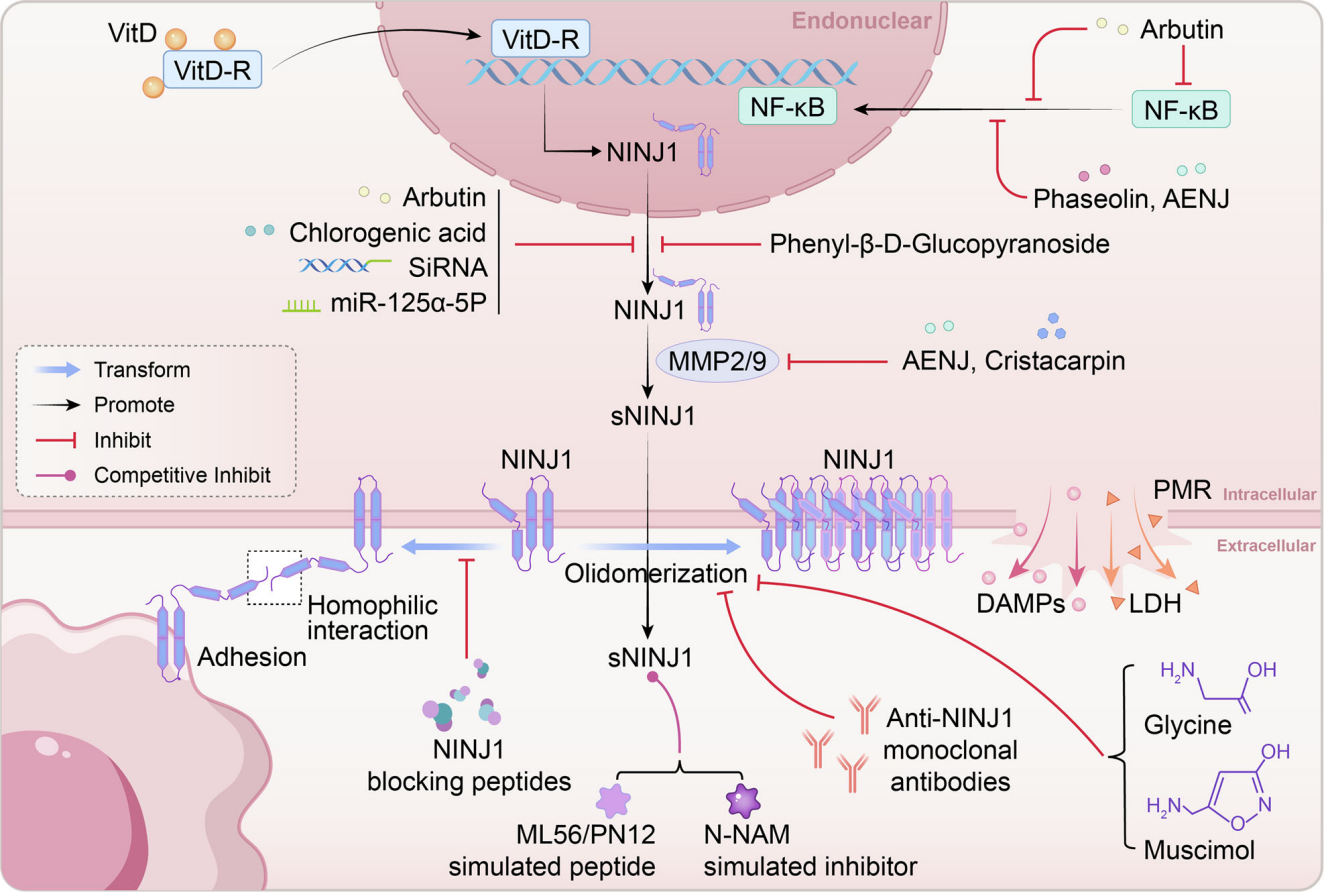
Strategic therapeutic targeting of distinct NINJ1 functional forms. This schematic summarizes representative therapeutic agents based on their primary mechanism.

Therefore, the choice between inhibition and modulation hinges on a precise understanding of which molecular form of NINJ1 is the key driver in a specific pathological context. The following sections summarize current agents within this conceptual framework (Fig. 5).

### Natural compounds

Multiple natural products have been found to effectively regulate the expression and function of NINJ1. Among them, natural polyphenols (including Arbutin [84] and Chlorogenic acid [85]), the flavonoid Phaseolin [86], the glycoside Phenyl-β-D-Glucopyranoside [87], and the marine natural product aqueous extract of Nomura’s jellyfish (AENJ) [88] all downregulate NINJ1 expression by blocking the nuclear translocation and transcriptional activity of NF-κB, indicating that the NF-κB signaling pathway is a key upstream mechanism regulating NINJ1 expression. Additionally, Vitamin D, acting as a steroid hormone, directly regulates NINJ1 expression through its receptor (VDR), identifying NINJ1 as a primary target of the vitamin D signaling pathway [89, 90].

It is particularly noteworthy that compounds such as Phaseolin, Phenyl-β-D-Glucopyranoside, and AENJ can also inhibit the activity of matrix metalloproteinases (MMPs) [86–88]. Given that NINJ1 is itself a substrate for cleavage by MMP-9, this mechanism suggests these compounds likely function primarily by interfering with the generation of the sNINJ1 and its mediated inflammatory signaling, rather than directly affecting the NINJ1 oligomers involved in PMR.

In contrast, the amino acid Glycine acts by directly inhibiting the oligomerization of NINJ1 [91]. This unique mechanism allows it to specifically block the final stage of PMR during pyroptosis, providing distinct value for controlling excessive inflammatory responses.

### Small molecule compounds

Synthetic small molecules offer more precise tools for targeting NINJ1. DMHCA forms a high-affinity binding with NINJ1, demonstrating good target specificity [92]. The isoprenylated dihydrochalcone Cristacarpin effectively reduces macrophage adhesion by downregulating the expression of both NINJ1 and MMP [93]. The GABA receptor agonist Muscimol, by blocking NINJ1 oligomerization, inhibits the final stage of PMR [94], providing a new strategy for controlling pyroptosis.

### Antibodies and targeted peptides

This class of therapeutic agents exhibits high specificity. Among the competitive peptide segments, the N-NAM mimetic inhibitor simulates the N-terminal adhesion motif of NINJ1, effectively inhibiting NINJ1-mediated cell migration and inflammatory signaling [81]. The ML56/PN12 mimetic peptide simulates sNINJ1, inhibiting pro-inflammatory gene expression in macrophages and the transendothelial migration of monocytes, thereby alleviating the progression of atherosclerosis [36]. Neutralizing antibodies show therapeutic potential in mitigating tissue damage by blocking NINJ1 oligomerization [57, 95, 96]. Furthermore, the microRNA miR-125a-5p effectively suppresses inflammatory responses and the progression of diabetic retinopathy by reducing NINJ1 expression [97].

## Conclusions and opportunities

The understanding of NINJ1’s biological roles has undergone a fundamental transformation. While initially characterized as a mediator of homophilic cell adhesion and a source of the chemokine-like sNINJ1 generated by MMP-9 cleavage, its identity was fundamentally expanded by the seminal 2021 discovery establishing NINJ1 oligomerization as the executor of PMR. This critical new dimension complements rather than negates its traditional functions, presenting the central challenge of deciphering how these diverse roles—adhesion, chemotactic signaling, and membrane disintegration—coexist and are regulated within a single molecular entity.

We propose that NINJ1’s functional diversity is intrinsically linked to its distinct molecular forms. The homophilic states primarily mediate its cell adhesion functions under homeostatic conditions. Proteolytic cleavage yields the sNINJ1, which acts as a chemokine to regulate cell migration in processes like inflammation and angiogenesis. Finally, the membrane-embedded oligomeric form serves as the terminal effector in cell death by executing membrane rupture. This “form dictates function” framework shifts the research focus from vague context-dependency toward fundamental regulatory questions: what specific intracellular signals and molecular events determine which functional form of NINJ1 is engaged at a given time and cellular location?

Our systematic review delineates the functional diversity of NINJ1 across multiple pathophysiological processes. The seemingly paradoxical observations—including its dual roles in opposing processes such as cell death versus survival, inflammation promotion versus suppression, and tissue destruction versus repair—collectively reveal NINJ1’s essence as a complex regulatory node. Comprehensive analysis indicates that NINJ1’s functional output is governed by four interrelated regulatory dimensions.

Functional specificity is primarily determined by molecular heterogeneity. NINJ1 achieves functional diversification through distinct molecular states: the full-length membrane protein mediates cell adhesion, the MMP-9-derived sNINJ1 exhibits chemotactic activity, while the activated oligomeric form executes membrane rupture. The contrasting protective role of sNINJ1 in atherosclerosis models versus the pathogenic function of full-length NINJ1 in diabetic vasculopathy confirms the direct correlation between molecular form and functional outcome. This finding suggests that interventional strategies targeting NINJ1 must account for the functional states of its specific molecular forms.

Cellular context dictates functional differentiation, as NINJ1’s biological effects strictly depend on the expressing cell type. It maintains vascular stability in pericytes, mediates inflammatory responses and cell death in macrophages, and promotes structural repair in neurons. The contradictory observations in inflammatory bowel disease models—where global knockout exacerbates disease while myeloid-specific knockout ameliorates it—demonstrate the decisive influence of cell type on NINJ1 function, indicating its participation in distinct signaling networks across different cell populations.

Temporal dynamics during pathological progression enable functional switching. NINJ1 exhibits spatiotemporal characteristics, with PMR serving host defense in localized infections while causing organ damage in systemic infections. Similarly, in cerebral ischemia models, NINJ1 function transitions from acute injury effects to potential repair roles during recovery, necessitating stage-specific interventional strategies.

Genetic background reprograms functional properties, as specific genetic environments can reconfigure NINJ1’s functional characteristics. The decisive impact of p53 status—where NINJ1 exhibits pro-tumorigenic function in wild-type p53 backgrounds but tumor-suppressive activity in p53-deficient contexts—provides compelling evidence for this reprogramming. The contradictory correlations between NINJ1 expression and prognosis across different cancer types further support the modifying effect of genetic background on NINJ1 function.

Another central unresolved question concerns the precise mechanism of NINJ1 activation. The molecular details of how NINJ1 transitions from a resting dimeric state to an active oligomeric complex capable of executing PMR remain largely undefined. Key questions include the exact conformational changes that drive this transition and whether NINJ1 autoinhibits its own pore-forming activity through an intramolecular mechanism—potentially analogous to the autoinhibitory structures observed in gasdermin proteins—that is relieved upon receiving a specific activation signal. Elucidating the atomic-level details of this conformational switch is fundamental to understanding the essential nature of its activation.

Furthermore, the upstream signaling pathways that regulate NINJ1 remain incompletely characterized. Beyond its established role in cell death, it is critical to determine which other physiological or pathological stimuli—such as mechanical stress, metabolic pressure, or specific cytokines—can modulate NINJ1. More importantly, how does NINJ1 perceive these diverse signals? A fundamental unanswered question is whether NINJ1 itself possesses receptor-like capabilities or engages with as-yet-unidentified specific ligands or interacting proteins to initiate downstream signaling cascades.

In the realm of biomarkers, sNINJ1 demonstrates unique translational potential. Recent studies indicate that elevated NINJ1 expression in monocytes/macrophages correlates positively with disease severity and prognosis in severe influenza A virus infection and COVID-19, suggesting sNINJ1 could serve as a universal biomarker for critical viral pneumonia [67]. Given NINJ1’s role as a common terminal effector in multiple lytic cell death pathways, its detection in blood or local lavage fluids holds promise for early warning, precise staging, and therapeutic monitoring in inflammatory conditions characterized by extensive cell death, such as sepsis and autoimmune diseases. This approach offers a distinct advantage: by reflecting the final stage of cellular disintegration, sNINJ1 may provide a more direct measure of tissue damage than upstream signaling molecules.

Capitalizing on the “form-determines-function” principle, developing therapeutics that target specific NINJ1 conformations is crucial for precision medicine. Key future directions include: developing compounds that specifically inhibit NINJ1 oligomerization (e.g., Glycine and its derivatives) to block PMR without compromising its adhesive role; designing inhibitors that disrupt MMP-9-mediated cleavage of NINJ1 to control sNINJ1 generation and its chemotactic activity; and employing neutralizing antibodies against the membrane-bound form or using competitive peptides (e.g., ML56/PN12) that mimic sNINJ1 for functional modulation. Such targeted interventions aim to maximally preserve NINJ1’s physiological functions while suppressing its pathological effects, thereby significantly improving therapeutic safety.

Given the complexity and redundancy of cell death pathways, combination therapies present a promising avenue. For instance, in cancer treatment, combining NINJ1 inhibitors with GSDMD inhibitors or immune checkpoint blockade could synergistically regulate cell death and immune activation for more effective tumor control. In sepsis, co-administration of NINJ1 inhibitors and anti-inflammatory agents might simultaneously mitigate primary cellular damage and subsequent inflammatory storms, achieving a dual therapeutic effect.

However, NINJ1-targeted therapies face significant challenges. Systemic inhibition of NINJ1 may compromise host defense against infections, as evidenced by its protective role in Citrobacter rodentium models [65]. Potential solutions include localized drug delivery strategies—such as inhalable formulations for pulmonary diseases or intrathecal administration for neurological disorders—to achieve high local concentrations at disease sites while minimizing systemic exposure. Alternatively, “dynamic regulation” employing transient NINJ1 inhibition during the acute inflammatory phase, followed by its release during recovery, could balance pathogen control and tissue repair.

However, translating this “form-determines-function” principle into safe and effective therapies presents formidable challenges, the foremost being the preservation of host defense. The essential role of NINJ1-mediated PMR in containing bacterial infections, such as Citrobacter rodentium, creates a fundamental therapeutic paradox: systemic inhibition risks increasing infection susceptibility. This necessitates a highly contextual risk-benefit assessment, where intervention is rational primarily in sterile or dysregulated inflammatory contexts (e.g., sterile tissue injury, autoimmunity) rather than during active infection. Consequently, future therapeutic development must move beyond speculative targeting to establish clear criteria for intervention. A precision strategy must be guided by the pathogenic NINJ1 form: inhibition of the oligomeric pore is logical for acute, lytic cell death-driven pathologies; modulation of sNINJ1 generation or signaling is preferable for chronic inflammatory diseases where its chemokine function dominates; and blockade of adhesive function could specifically target leukocyte trafficking. Achieving this specificity—developing agents that distinguish between NINJ1’s molecular forms—and solving the associated delivery challenges to minimize systemic impact, constitute the critical path forward for NINJ1-targeted medicine.

This raises the classic challenge inherent to all single-gene targeted interventions: the dual problems of drug delivery and specificity. Valuable insights can be drawn from mature technologies in other fields. For tissue-specific targeting, strategies like antisense oligonucleotide technology offer pathways to circumvent the poor specificity and off-target effects that often plague conventional protein-targeting drugs [98]. Similarly, for cell-type-specific delivery, platforms such as antibody-oligonucleotide conjugates or ligand-functionalized nanoparticles can be leveraged to precisely direct NINJ1 inhibitors to specific cell populations like macrophages [99, 100]. Furthermore, advanced delivery systems, including lipid nanoparticles and biomimetic membrane-coated nanoparticles, have demonstrated significant progress in enhancing drug solubility, prolonging circulation half-life, and improving intracellular delivery efficiency. These systems hold great promise for optimizing the pharmacokinetic profile of future NINJ1-targeted therapies [101, 102].
